# Transcriptional Effects of E3 Ligase *Atrogin-1*/*MAFbx* on Apoptosis, Hypertrophy and Inflammation in Neonatal Rat Cardiomyocytes

**DOI:** 10.1371/journal.pone.0053831

**Published:** 2013-01-15

**Authors:** Yong Zeng, Hong-Xia Wang, Shu-Bin Guo, Hui Yang, Xiang-Jun Zeng, Quan Fang, Chao-Shu Tang, Jie Du, Hui-Hua Li

**Affiliations:** 1 Department of Cardiology, Peking Union Medical Hospital, Beijing, China; 2 The Key Laboratory of Remodeling-related Cardiovascular Diseases, Department of Pathology and Pathophysiology, School of Basic Medical Sciences, Capital Medical University, Beijing, China; 3 Department of Emergency Medicine, Peking Union Medical Hospital, Beijing, China; 4 The Key Laboratory of Remodeling-related Cardiovascular Diseases, Laboratory of Vascular Biology, Beijing Institute of Heart Lung and Blood Vessel Diseases, Beijing Anzhen Hospital Affiliated the Capital Medical University, Beijing, China; University of Western Ontario, Canada

## Abstract

*Atrogin-1*/MAFbx is an ubiquitin E3 ligase that regulates myocardial structure and function through the ubiquitin-dependent protein modification. However, little is known about the effect of *atrogin-1* activation on the gene expression changes in cardiomyocytes. Neonatal rat cardiomyocytes were infected with adenovirus *atrogin-1* (Ad-*atrogin-1*) or GFP control (Ad-GFP) for 24 hours. The gene expression profiles were compared with microarray analysis. 314 genes were identified as differentially expressed by overexpression of *atrogin-1*, of which 222 were up-regulated and 92 were down-regulated. *Atrogin-1* overexpression significantly modulated the expression of genes in 30 main functional categories, most genes clustered around the regulation of cell death, proliferation, inflammation, metabolism and cardiomyoctye structure and function. Moreover, overexpression of *atrogin-1* significantly inhibited cardiomyocyte survival, hypertrophy and inflammation under basal condition or in response to lipopolysaccharide (LPS). In contrast, knockdown of atrogin-1 by siRNA had opposite effects. The mechanisms underlying these effects were associated with inhibition of MAPK (ERK1/2, JNK1/2 and p38) and NF-κB signaling pathways. In conclusion, the present microarray analysis reveals previously unappreciated *atrogin-1* regulation of genes that could contribute to the effects of *atrogin-1* on cardiomyocyte survival, hypertrophy and inflammation in response to endotoxin, and may provide novel insight into how *atrogin-1* modulates the programming of cardiac muscle gene expression.

## Introduction

Heart failure (HF) is the final and common pathway for various cardiovascular diseases. Lipopolysaccharide (LPS) from gram negative bacteria is one of the most common causes of inflammation and innate immunity through Toll-like receptor-4 (TLR-4) [1], [2]. Several studies demonstrated that LPS directly induces cardiomyocyte hypertrophy, apoptosis and depresses contractility, ultimately leading to cardiomyopathy and congestive heart failure [3]. Accumulating evidence has shown that cardiac muscle mass loss, because of increased rate of protein degradation and cellular death, represents a critical pathogenic event in HF [4]. Disturbances in the ubiquitin-proteasome system are thought to be involved in the development of various cardiovascular diseases, including HF, cardiac infarction and atherosclerosis [4]–[8]. Recently, a study has identified two novel ubiquitin E3 ligases, *atrogin-1*/*MAFbx* and *MuRF1*, which function as negative regulator of muscle cell size in vitro and in vivo [5], [6], [9]–[12].

*Atrogin-1* is an F-box protein selectively expressed in cardiac and skeletal muscle tissues, and is known to be up-regulated markedly in skeletal muscle in a variety of models of catabolic states, including oxidative stress, fasting, cancer, sepsis, heart failure [8]–[10], [13], [14]. Importantly, *atrogin-1* associates with Skp1, Cul1 and Roc1 to form an SKP1-CUL1-F-box (SCF)-type ubiquitin ligase [5], [6], [10], [15]. Overexpression of *atrogin-1* in skeletal myotubes leads to atrophy, whereas *atrogin-1* deficiency results in marked resistance to skeletal muscle denervation atrophy [10]. Moreover, *atrogin-1* confers SCF complex specificity by directly targeting many substrates including calcineurin, initiation factor eIF3-f, Myo D, and mitogen-activated protein kinase phosphatase-1 (MKP-1) for proteasome-dependent degradation, leading to negative regulation of muscle cell size and cardiomyocte survival [5], [16]–[19]. However, We have recently described a novel role for atrogin-1 as a co-activator of FOXO1/3a through lysine 63-linked polyubiquitination, thereby inhibiting Akt-dependent physiologic hypertrophy [6]. These results suggest that *atrogin-1* plays a pivotal role in muscle atrophy, cardiac hypertrophy and cardiomyocyte apoptosis.

Although it is well known that upstream proteins including Foxo1/3a that activate *atrogin-1* transcription and enhance its activity for protein degradation are required for heart failure [15], [20], [21], little is known about how *atrogin-1* contributes cardiomyocyte apoptosis, proliferation and hypertrophy through regulating gene expression at the transcriptional level. To gain insight into the early molecular events associated with *atrogin-1*-mediated cardiomyocyte apoptosis, hypertrophy and inflammation, we used primary neonatal rat cardiomyocytes as a model to investigate the effects of *atrogin-1* on cardiac muscle gene expression, and confirmed the results by real-time quantitative PCR analysis. Our results showed that *atrogin-1* overexpression modulated the expression of many genes involved in the regulation of diverse biological functions, including cell survival, proliferation, inflammation, cell metabolism and cardiac hypertrophy. Importantly, *atrogin-1* inhibits endotoxin LPS-induced cardiomyocyte apoptosis, hypertrophy and inflammation through mitogen activated protein kinases (MAPKs) and NF-κB signaling pathways. We believe that such a comprehensive analysis of gene expression profile in neonatal rat cardiomyocytes may identify novel targets for drug discovery in the intervention of the progression of cardiac dysfunction.

## Results

### Effects of *atrogin-1* overexpression on gene expression profiles in neonatal rat cardiomyocytes

**Figure 1:**
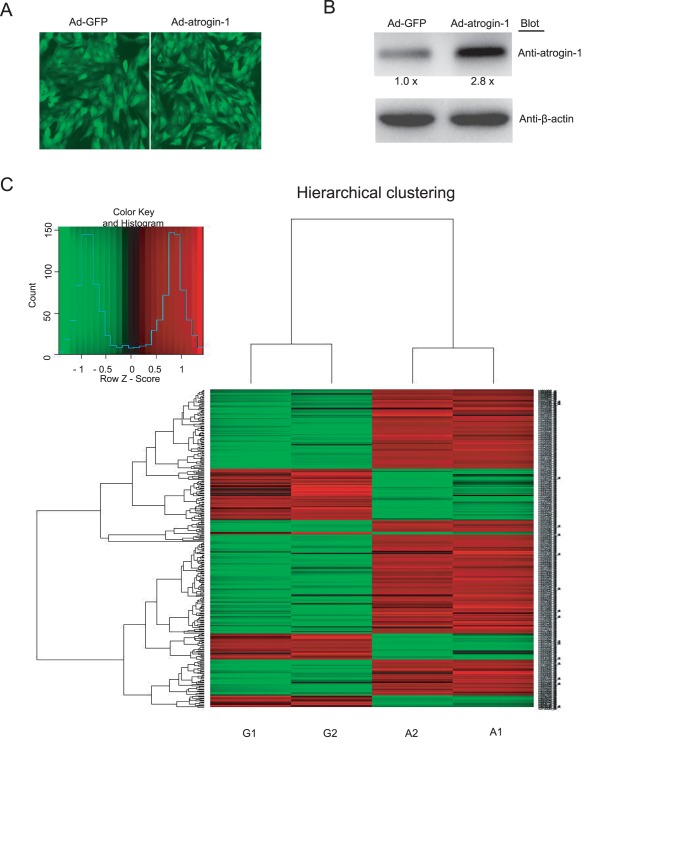
Infection of adenovirus *atrogin-1* and microarray analysis. **A.** Neonatal rat cardiomyocytes were infected with adenovirus green fluorescent protein (GFP) control (Ad-GFP) or *atrogin-1* (Ad-*atrogin-1*). The infection efficiency was visualized for GFP 24 hours later using fluorescence microscopy (Magnification, ×400). **B.** The levels of *atrogin-1* protein were determined by Western blot analysis with anti-*atrogin-1* antibody, using β-actin as the internal control. Quantitative analysis of protein bands was shown (n = 3). **C.** Hierarchical clustering depicting expression profiles of differentially expressed genes in Ad-*atrogin-1* (A1 and A2) and Ad-control (G1 and G2) groups. Data from individual sample are shown. A subset of genes displays significant expression changes at ≥2-fold or ≤−2-fold. Gene expression levels are shown as color variations (red: up-regulated expression; green: down-regulated expression).

To identify genes differentially regulated by *atrogin-1* in cardiomyocytes, we examined the changes in the gene expression profiles of cardiomyocytes infected by adenovirus atrogin-1 (Ad-*atrogin-1*) and GFP control (Ad-GFP) using DNA microarray assay. After 24 hours, the infection efficiency was more than 95% as characterized by GFP, the level of atrogin-1 protein in cardiomyocytes was increased by 2.8-fold compared to Ad-GFP control (Figure 1A and B). Moreover, we found that overexpression of *atrogin-1* in cultured cardiomyocytes leads to 314 genes being significantly regulated when compared to control group. Of them, 222 were up-regulated and 92 down-regulated by *atrogin-1* overexpression. A hierachical clustering on the complete set of data of present genes was performed as described [22]. The analysis showed a clear separation of the Ad-GFP control (G) and overexpressed *atrogin-1* (A) groups, when a clustering was performed on a subset of genes that displayed different expression with fold changes ≥2-fold or ≤−2-fold (Figure 1C).

### Validation of microarray analysis with qRT-PCR

**Figure 2:**
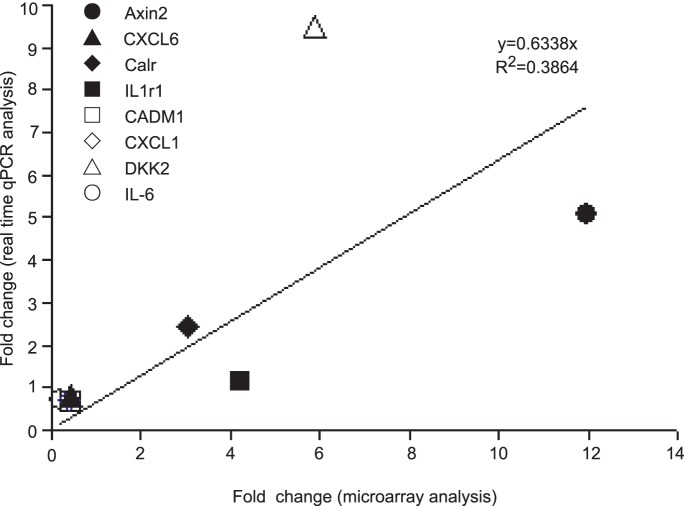
Scatter plot of relative changes in gene expression as determined by microarray analysis and by qRT-PCR. Eight genes were analyzed: IL-6, DKK2, Calr, Cxcl6, Cxcl1, Axin2, IL-1r1, and Cadm1. Each symbol represents the fold change of the respective gene in ad-*atrogin-1* group over Ad-GFP control.

To examine the reliability of microarray results, we performed qRT-PCR analysis for a set of eight genes including Axin2, Cxcl6, Calr, IL-1r1, Cadm1, Cxcl1, Dkk2 and IL-6, which were differentially expressed in the microarray assay. Scatter plot analysis of the relative changes in expression as determined by qRT-PCR and microarray revealed a good correlation (Figure 2).

**Figure 3:**
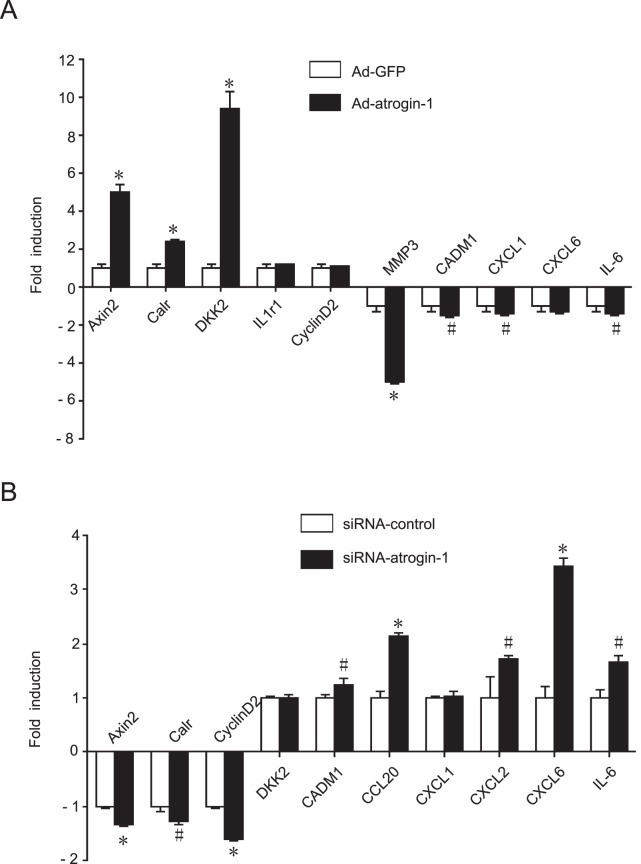
qRT-PCR analysis of microarray data. Neonatal rat cardiomyocytes were infected with Ad-*atrogin-1* or Ad-GFP (**A**), Ad-siRNA-*atrogin-1* or Ad-siRNA-control (**B**) for 24 hours. Total RNA was extracted and qRT-PCR analysis was performed in triplicate using specific oligonucleotides primers. The differences in gene expression levels were statistically significant. Data represent the mean±SEM (n = 3 per group). ^#^ *P*<0.05; *P<0.001 vs. Ad-GFP control.

To validate the microarray data, we performed qRT-PCR for a set of 10 genes that were differentially expressed in *atrogin-1*-infected cardiomyocytes. 7 of 10 genes showed similar expression ratios between qRT-PCR data and microarray results. Overexpressed *atrogin-1* significantly induced the transcription of cell proliferation-related genes including the Axin2, Calr and DKK2, and down-regulated the transcriptional level of cardiac remodeling-related gene MMP3, adhesion-related gene Cadm1 and chemotaxis-related genes including Cxcl1, Cxcl6 and IL-6 compared to Ad-GFP control (Figure 3A). In contrast, knockdown of *atrogin-1* by siRNA significantly decreased the transcriptional level of Calr, Axin2 and Ccnd2, and up-regulated the transcriptional level of Cadm1, Ccl20, Cxcl1, Cxcl2, Cxcl6 and IL-6 compared to siRNA-control (Figure 3B). Collectively, these results further provide evidence to support the validity and quality of the microarray data.

### Functional characterization of the atrogin-1-regulated genes

Next, we clustered atrogin-1-regulated genes into functional groups using gene annotation information from the Affymetrix database. Our data showed that the most prominently enriched biological process in the differentially atrogin-1-regulated genes was cell apoptosis, proliferation, metabolism, hypertrophy and inflammation.

### Genes involved in cell apoptosis

Twenty-two genes were differentially regulated by atrogin-1 overexpression (Table 1). Eight of them including Csf2 [23]–[25], IL-6 [26]–[28] and Aldh1a3 [29] were down-regulated, which were involved in negative regulation of apoptosis. Conversely, fourteen of them including Msx2 [30] and Axin2 [31] were up-regulated, which mediated positive regulation of apoptosis.

**Table 1:** GO:0008219 cell apoptosis and death.

Gene Symbol	Gene Title	Fold change	P value
Rab27a	RAB27A, member RAS oncogene family	5.69	0.0011
Prkcz	protein kinase C, zeta	3.66	4.0E-05
Msx2	msh homeobox 2	3.59	0.027
Herpud1	homocysteine-inducible, endoplasmic reticulum stress-inducible, ubiquitin -like d	3.47	5.7E-05
Dnajb9	DnaJ (Hsp40) homolog, subfamily B, member 9	3.27	6.1E-05
Hspa5	heat shock protein 5	3.19	2.8E-04
Tcf7	transcription factor 7, T-cell specific	2.82	0.0017
Pdia3	protein disulfide isomerase family A, member 3	2.69	1.3E-05
Pdia2	protein disulfide isomerase famil	2.33	0.0039
Sels	selenoprotein S	2.29	1.5E-04
Eif2ak3	eukaryotic translation initiation factor 2 alpha kinase 3	2.14	3.1E-04
Gclc	glutamate-cysteine ligase, catalytic subunit	2.05	0.018
Psen2	presenilin 2	−2.02	7.8E-04
Aldh1a3	aldehyde dehydrogenase 1 family, member A3	−2.04	0.05
Mapt	microtubule-associated protein tau	−2.17	0.027
Cadm1	cell adhesion molecule 1	−2.34	0.0026
Ptgs2	prostaglandin-endoperoxide synthase 2	−3.56	0.020
Csf2	colony stimulating factor 2 (granulocyte-macrophage)	−3.78	0.042
Il-6	interleukin 6	−4.83	0.006

### Genes involved in cell proliferation

One of the most significant overrepresented terms of biological process was cell proliferation in Ad-*atrogin-1* group compared to Ad-GFP control group. Eighteen genes were differentially regulated, which are involved in cell proliferation (Table 2). Of them, thirteen genes such as Ptgs2 [32] were down-regulated, which mediated positive regulation of cell proliferation. In contrast, seven genes such as Axin2 [31], Msx2 [30] and Prkcz (PKCε) [33] involved in negative regulation of cell proliferation and cell cycle arrest were up-regulated.

**Table 2:** GO:0008283 cell proliferation.

Gene Symbol	Gene Title	Fold change	P value
Axin2	axin2	12.2	6.6E-06
Prkcz	protein kinase C, zeta	3.66	4.0E-05
Msx2	msh homeobox 2	3.59	0.027
Abcc4	ATP-binding cassette, sub-family C (CFTR/MRP), member 4	3.20	1.3E-04
Calr	calreticulin	3.05	1.2E-04
Mycn	v-myc myelocytomatosis viral related oncogene, neuroblastoma derived (avian)	2.85	1.5E-04
Tcf7	transcription factor 7, T-cell specific	2.82	0.0017
Fgf9	fibroblast growth factor 9	2.42	0.0038
Ccnd2	cyclin D2	2.21	0.0067
Cdk5rap3	CDK5 regulatory subunit associated protein 3	2.03	0.0021
Klf5	Kruppel-like factor 5	−2.17	0.013
Cadm1	cell adhesion molecule 1	−2.34	0.0026
Ptges	prostaglandin E synthase	−3.21	0.05
Ptgs2	prostaglandin-endoperoxide synthase 2	−3.56	0.020
Csf2	colony stimulating factor 2 (granulocyte-macrophage)	−3.78	0.04

### Genes involved in metabolisms

Metabolic pathways was a crucial signaling pathway through which sixteen genes were significantly enriched (Table 3). Twelve genes such as Ptgis [34] and Pycr1 [35] were up-regulated. Conversely, four genes including Ckmt1 and Ptges were down-regulated, which mediates negative regulation of mitochondrial function [36].

**Table 3:** Metabolic pathways.

Gene Symbol	Gene Title	Fold change	P value
Isyna1	inositol-3-phosphate synthase 1	4.09	3.8E-05
Gmppb	GDP-mannose pyrophosphorylase B	3.62	2.6E-06
Pycr1	pyrroline-5-carboxylate reductase 1	3.15	0.004
LOC684425	similar to Adenylosuccinate synthetase isozyme 1	2.57	0.05
Alg12	asparagine-linked glycosylation 12 homolog	2.27	4.4E-05
Nans	N-acetylneuraminic acid synthase	2.26	2.2E-04
Oat	ornithine aminotransferase (gyrate atrophy)	2.25	3.8E-04
Rpn1	ribophorin I	2.13	7.5E-06
Slc33a1	Solute carrier family 33	2.09	5.9E-04
Stt3a	STT3, subunit of the oligosaccharyltransferase complex, homolog A	2.07	0.002
Gclc	glutamate-cysteine ligase, catalytic subunit	2.05	0.018
Bcat2	branched chain aminotransferase 2, mitochondrial	2.01	0.004
Aldh1a3	aldehyde dehydrogenase 1 family, member A3	−2.04	0.05
Ptgis	prostaglandin I2 (prostacyclin) synthase	−2.10	0.016
Ptges	prostaglandin E synthase	−3.21	0.05
Ckmt1	creatine kinase, mitochondrial 1, ubiquitous	−3.35	0.0035
Ptgs2	prostaglandin-endoperoxide synthase 2	−3.56	0.02

### Genes involved in cell development and hypertrophic cardiomyopathy

Seventeen genes were closely related to the regulation of cell development (Table 4). Thirteen of them such as Myh6 (α-MHC) [37], Bhlha15 (Mist1) [38] and Serca2 (ATP2A2) [39], [40] and Prkcz that are involved in cell maturation, sarcomere organization and hypertrophy were up-regulated. Four of them such as Foxa2 [41] and ITGA3 were down-regulated, which mediate regulation of gene expression in response to insulin stimulus and cell differentiation.

**Table 4:** GO:0048468 cell development and hypertrophy.

Gene Symbol	Gene Title	Fold change	P value
Krt19	keratin 19	13.9	0.0035
Bhlha15	basic helix-loop-helix family, member a15	4.27	1.1E-04
Calr	calreticulin	3.05	1.2E-04
Lef1	lymphoid enhancer binding factor 1	2.98	1.7E-04
Csrp2	cysteine and glycine-rich protein 2	2.36	0.0021
Ccnd2	cyclin D2	2.21	0.0067
Eif2ak3	eukaryotic translation initiation factor 2 alpha kinase 3	2.14	3.1E-04
Hook1	hook homolog 1 (Drosophila)	2.13	6.3E-04
Xbp1	X-box binding protein 1	2.07	4.1E-05
Lppr4	plasticity related gene 1	2.05	0.018
Cdk5rap3	CDK5 regulatory subunit associated protein 3	2.03	0.0021
Myh6	myosin, heavy chain 6, cardiac muscle, alpha	2.02	0.05
Mapt	microtubule-associated protein tau	−2.17	0.027
Itga3	Integrin alpha 3	−2.18	1.6E-05
Foxa2	forkhead box A2	−5.28	2.0E-05

### Genes involved in inflammation

Response to inflammation and stress was a prominently overrepresented GO term in which thirty-five genes were involved (Table 5), nineteen of them involved in inflammatory response were significantly down-regulated by overexpression of *atrogin-1*, these genes include IL-6 [42], Ptger3 [43], Lbp [44], Ptgs2 (Cox-2) [45] and chemokines such as Cxcl1, Cxcl2, Cxcl3, Ccl20 and Cxcl6 [46], [47]. However, sixteen of them such as IL-1r1 was significantly up-regulated [48].

**Table 5:** GO:0006950 response to inflammation.

Gene Symbol	Gene Title	Fold change	P value
Dnajc3	DnaJ (Hsp40) homolog, subfamily C, member 3	6.77	4.2E-05
Rab27a	Similar to RIKEN cDNA 1110059E24	5.69	0.0011
Hyou1	hypoxia up-regulated 1	5.48	5.7E-06
Il1r1	interleukin 1 receptor, type I	4.16	0.001
Armet	arginine-rich, mutated in early stage tumors	3.81	1.1E-04
Herpud1	homocysteine-inducible, endoplasmic reticulum stress-inducible, ubiquitin-like d	3.46	5.7E-05
Dnajb9	DnaJ (Hsp40) homolog, subfamily B, member 9	3.27	6.1E-05
Hspa5	heat shock protein 5	3.19	2.8E-04
Pdia2	protein disulfide isomerase famil	2.33	0.004
Sels	selenoprotein S	2.29	1.5E-04
Eif2ak3	eukaryotic translation initiation factor 2 alpha kinase 3	2.15	3.1E-04
Hspb7	heat shock protein family, member 7 (cardiovascular)	2.12	3.8E-04
Xbp1	X-box binding protein 1	2.07	4.1E-05
Gclc	glutamate-cysteine ligase, catalytic subunit	2.05	0.018
Txndc4	thioredoxin domain containing 4 (endoplasmic reticulum)	2.03	7.4E-05
Serp1	stress-associated endoplasmic reticulum protein 1	2.02	0.0019
Psen2	presenilin 2	−2.03	7.8E-04
Lbp	lipopolysaccharide binding protein	−2.10	0.05
Ptgis	prostaglandin I2 (prostacyclin) synthase	−2.10	0.016
Cxcl3	chemokine (C-X-C motif) ligand 3	−2.13	0.05
Asf1a	ASF1 anti-silencing function 1 homolog A (S. cerevisiae)	−2.18	4.0E-4
Cxcl6	chemokine (C-X-C motif) ligand 6	−2.25	0.045
Ptges	prostaglandin E synthase	−2.25	0.05
Cadm1	cell adhesion molecule 1	−2.34	0.0026
Tfpi2	tissue factor pathway inhibitor 2	−2.37	0.05
Hpx	hemopexin	−2.45	0.02
Ptger3	Prostaglandin E receptor 3 (subtype EP3)	−2.50	0.016
Cxcl1	chemokine (C-X-C motif) ligand 1	−2.50	0.016
Cxcl2	chemokine (C-X-C motif) ligand 2	−2.60	0.05
Reg3g	regenerating islet-derived 3 gamma	−2.60	0.05
Ccl20	chemokine (C-C motif) ligand 20	−3.17	0.006
Ptgs2	prostaglandin-endoperoxide synthase 2	−3.56	0.02
Il-6	interleukin 6	−4.83	0.006
Serpinb2	serine (or cysteine) peptidase inhibitor, clade B, member 2	−5.45	0.017

### Effects of atrogin-1 on cardiomyocyte apoptosis, hypertrophy and inflammation

**Figure 4:**
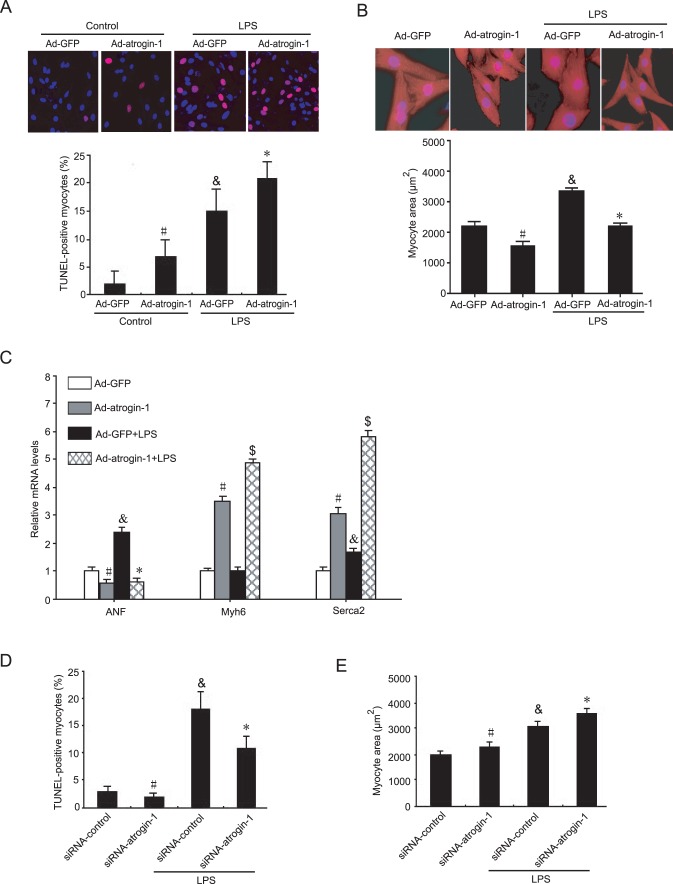
Effects of *atrogin-1* overexpression on cardiomyocyte apoptosis and hypertrophy. Neonatal rat cardiomyocytes were infected with Ad-GFP or Ad-*atrogin-1*-GFP for 24 h and then treated with LPS (1 µg/ml) for additional 24 hours. **A.** Apoptosis was detected and quantified using TUNEL assay (red), and nuclei were counterstained with DAPI (blue). A representative field is shown for each condition (top panels), Magnification, ×400. Quantitative analysis of TUNEL-positive cells from three independent experiments (bottom panels). **B.** The cells were fixed and stained with anti-α-actinin antibody followed by Alexa Fluor 568-conjugated goat anti-mouse IgG (red), and nuclei were stained with DAPI (blue). A representative field is shown for each condition (top panels), Magnification, ×200. Quantitative analysis of cell surface area (a minimum of 100 randomly chosen cells measured in each group) (bottom panels). Data represent the mean ± SEM (n = 3). **^#^** *P*<0.05, **^&^** *P*<0.01 vs. Ad-GFP; *P<0.05; ^$^ *P*<0.01 vs. Ad-GFP+LPS. **C.** The qRT-PCR analysis of ANF, Myh6 and serca2 mRNA expression was performed in triplicate using specific oligonucleotides primers. **D** and **E.** Neonatal rat cardiomyocytes were infected with Ad-siRNA-*atrogin-1* or Ad-siRNA-control for 24 hours. Analysis of apoptosis and cell surface area were performed as in A and B. Data represent the mean ± SEM (n = 3). **^#^** *P*<0.05, **^&^** *P*<0.01 vs. siRNA-control; *P<0.05 vs. siRNA-control +LPS.

**Figure 5:**
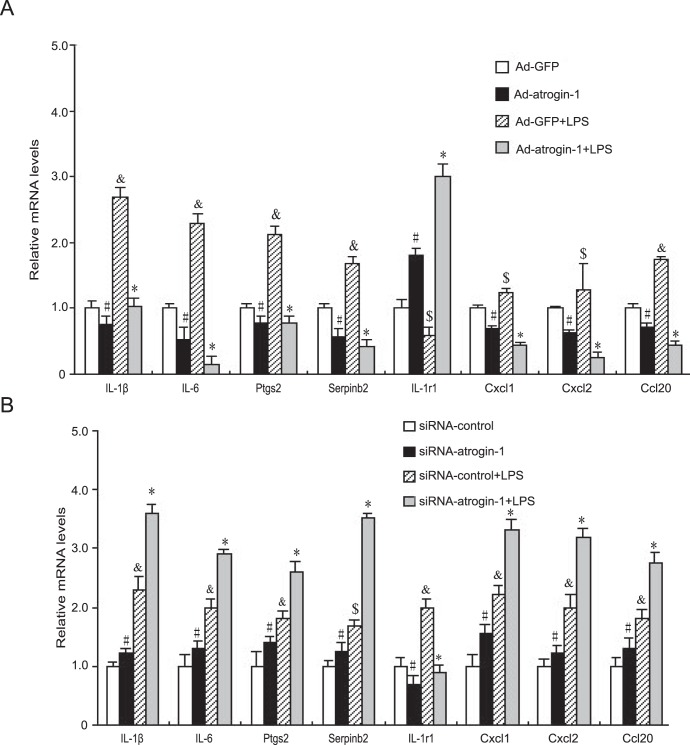
Effect of *atrogin-1* on inflammation. **A.** Neonatal rat cardiomyocytes were infected with Ad-GFP or Ad-*atrogin-1*-GFP for 24 h and then treated with LPS (1 µg/ml) for additional 24 hours. The qRT-PCR analysis of gene expression was performed in triplicate using specific oligonucleotides primers. Data represent the mean ± SEM. **^#^** *P*<0.05, **^&^** *P*<0.01, ^$^ *P*<0.05 vs. Ad-GFP; *P<0.01 vs. Ad-GFP+LPS. **B.** Neonatal rat cardiomyocytes were infected with Ad-siRNA-control or Ad-siRNA-*atrogin-1* for 24 h and then treated with LPS (1 µg/ml) for additional 24 hours. The qRT-PCR analysis of gene expression was performed as in A. Data represent the mean ± SEM. **^#^** *P*<0.05, **^&^** *P*<0.01, ^$^ *P*<0.05 vs. siRNA-control; *P<0.01 vs. Ad-siRNA+LPS.

Since genes differentially regulated by *atrogin-1* in cardiomyocytes have been implicated in cell apoptosis, hypertrophy and inflammation (Table 1, 4 and 5), which cause cardiac dysfunction and ultimately lead to heart failure, we then investigated whether overexpression of atrogin-1 influences cardiomyocyte apoptosis. Consistent with previous reports [18], *atrogin-1* overexpression significantly increased TUNEL-positive cardiomyocytes compared to Ad-GFP control (Figure 4A). After LPS stimulation, TUNEL-positive cardiomyocytes were significantly increased, and this effect was further enhanced by Ad-*atrogin-1* infection (Figure 4A). Moreover, consistent with our previous reports [5], [6], [49], *atrogin-1* overexpression markedly reduced cardiomyocyte surface areas under basal condition and in response to LPS stimulation (Figure 4B). qPCR analysis of hypertrophic markers (ANF, a-MHC and Serca2) further confirmed the effect of atrogin-1 on cardiomyocyte hypertrophy (Figure 4C). In contrast, knockdow of atrogin-1 by siRNA signaficantly reversed these effcts under basal condition or in response to LPS (Figure 4D and E). Finally, *atrogin-1* overexpression markedly inhibited the expression of proinflammatory-related genes (including IL-1β, IL-6, Ptgs2 and Serpinb2) and chemokines (Cxcl1, Cxcl2 and Ccl20) but up-regulated anti-inflammatory gene IL-1r1 expression compared to Ad-GFP control under basal condition or in response to LPS stimulation (Figure 5A), whereas these effects were reversed by knockdown of *atrogin-1* (Figure 5B). These results further confirmed the data from microarray analysis (Table 5). Collectively, these results indicate that increased expression of *atrogin-1* inhibits cardiomyocyte survival, hypertrophy and inflammation under basal condition or in response to LPS stimulation.

### Effects of atrogin-1 on MAPKs and NF-κB signaling pathways

**Figure 6:**
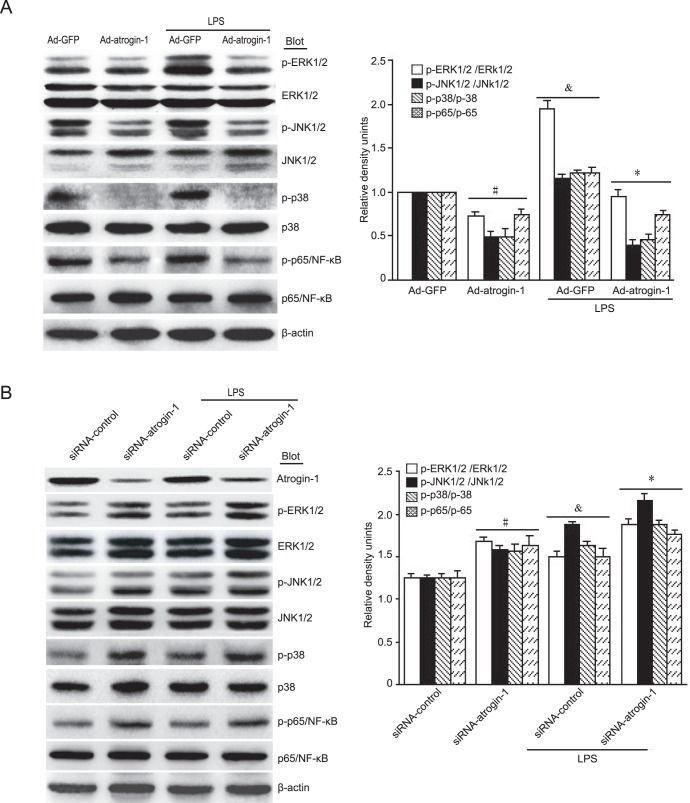
Effects of *atrogin-1* on MAPK and NF-κB signaling pathways. **A.** Neonatal rat cardiomyocytes were infected with Ad-GFP or Ad-atrogin-1-GFP for 24 h and then treated with LPS (1 µg/ml). The protein levels of total and phospho-ERK1/2, JNK1/2, p38 and p65/NF-κB were detected by Western blot analysis (left panels). Quantitative analysis of relative intensity of phosphorylated proteins was shown (right panel). Data represent the mean ± SEM (n = 3). **^#^** *P*<0.05, **^&^** *P*<0.05 vs. Ad-GFP; *P<0.01 vs. Ad-GFP+LPS. **B.** Neonatal rat cardiomyocytes were infected with adenovirus siRNA-control or siRNA-atrogin-1 and then treated with LPS (1 µg/ml). The protein levels were detected as in A (left panels). Quantitative analysis of relative intensity of phosphorylated proteins was shown (right panel). Data represent the mean ± SEM (n = 3). **^#^** *P*<0.05, **^&^** *P*<0.05 vs. siRNA-control; *P<0.05 vs. siRNA-control+LPS.

To elucidate the mechanisms for *atrogin-1* to regulate transcriptional gene expression, we examined the activation of two major signaling pathways of MAPK and NF-κB, which play critical roles in controlling the expression of apoptosis-, hypertrophy- and inflammation-related genes [50]. Our results showed that under saline treatment, overexpression of *atrogin-1* decreased the levels of ERK1/2, JNK1/2, p38 and p-65 phosphorylation protein compared to Ad-GFP control (Figure 6A). After LPS stimulation, the levels of ERK1/2, JNK1/2, p38 and p-65 phosphorylation were significantly increased compared to Ad-GFP control, However, LPS-induced effects were markedly attenuated by overexpression of *atrogin-1* in cardiomyocytes (Figure 5A). In contrast, depletion of *atrogin-1* by siRNA had opposite effects (Figure 6B). Together, these results suggest that MAPKs and NF-κB signaling pathways could be attributed to the effects of *atrogin-1* in cardiomyocytes under basal condition or after LPS treatment.

## Discussion

The present results demonstrate that *atrogin-1* activation results in the differential regulation of 314 genes in neonatal rat cardiomyocytes, of which 222 were up-regulated and 92 were down-regulated. Interestingly, the majority of differentially and highly expressed genes were involved in cell death, proliferation, inflammation, metabolism and cardiomyopathy. Moreover, increased expression of *atrogin-1* significantly inhibited caerdiomyocyte survival, hypertrophy and inflammation under basal condition or in response to LPS stimulation. The mechanisms underlying these effects were associated with inhibition of MAPK (ERK1/2, JNK1/2 and p38) and NF-κB signaling pathways.

Emerging evidences suggest that *atrogin-1* is a muscle-specific E3 ligase and is up-regulated by various of catabolic conditions [8]–[10], [12], [51]. Recent studies demonstrate that TNF-α, doxorubicin (DOX) and LPS can stimulate *atrogin-1* mRNA expression and ubiquitin conjugating activity in skeletal muscle through activation of p38 MAPK [51]–[53]. In contrast, AKT activation decreases *atrogin-1* expression through inhibition of FoxO transcriptional factors [54]. In addition, *atrogin-1* protein is degradated by proteasome via p38 MAPK signaling pathway [55]. These data indicate that multiple signaling pathways regulate atrogin-1 expression at different levels.

Apoptotic cell death in cardiac myocytes appears to be an early event and is well recognized to be responsible for several cardiovascular diseases such as HF and myocardial infarction [56]. LPS, one of the most common causes of inflammation, directly induces cardiomyocyte apoptosis and hypertrophy through calcineurin signaling pathway [3], [49], [51]. However, it is unclear that whether atrogin-1 contributes to LPS-induced cardiomyocyte apoptosis. Recently, our data show that *atrogin-1* plays an important role in regulating cardiomyocyte apoptosis. Overexpression of *atrogin-1* promotes ischemia/reperfusion (I/R)-induced cardiomyocyte apoptosis through activation of JNK signaling pathway [18]. However, the mechanisms by which *atrogin-1* promotes cardiomyocyte apoptosis remain to be elucidated. In this study, we examined the gene expression profiling regulated by *atrogin-1* overexpression in cardiomyocytes. Our microarray data showed that many genes involved in cell apoptosis and proliferation, including Csf2, IL-6, Ptgs2, Aldh1a3, Axin2, Msx2, Prkcz, Calr, and Pdia3 were differentially regulated (Table 1 and 2). Csf2 has been known to exert anti-apoptotic activity through the expression of Bcl-2 family proteins in neural progenitor cells via JAK/STAT5-Bcl-2 pathway [23]. Csf2 also abrogates ischemia and thus prevents cardiomyocyte death by neovascularization [24]. IL-6 activates JAK/STAT3 and phosphatidylinositol 3-kinase (PI3K) pathways, thereby promoting cardiomyocyte survival [27], [28]. Conversely, Axin2, Msx2, Calr and PKCε that mediate inhibition of cell proliferation and survival were up-regulated. Axin2, a most highly up-regulated gene (12.17-fold), is a negative regulator of the Wnt signaling pathway that promotes the phosphorylation and degradation of β-catenin, resulting in cardomyocyte survival and hypertrophy [31], [57]. PKCε can phosphorylate insulin receptor substrates (IRS) on serine residues impairing activation of PI3K in response to insulin [33], [58]. Loss of PKCε selectively impairs signaling through the B-cell receptor, resulting in inhibition of cell proliferation and survival, as well as defects in the activation of ERK and the transcription of NF-κB-dependent genes [59]. Consistent with these data, our results showed that increased expression of *atrogin-1* significantly promoted cardiomyocyte apoptosis under basal condition or in response to LPS stimulation (Figure 4).

Hypertrophy is a major contributor to cardiac dysfunction in human. Alterations of cardiac gene expression are central to ventricular dysfunction in human HF [5]. Our previous studies and others have demonstrated that *atrogin-1* plays a crucial role in skeletal and cardiac muscle plasticity in response to hypertrophic or atrophic stimuli [5], [6], [10], [18]. Accumulating evidences indicate that *atrogin-1*-dependent proteolysis of its substrates such as calcineurin, FOXOs, MyoD and eIF3-f could constitute the important events to regulate myocyte growth and hypertrophy [5], [6], [17], [19]. However, the detailed molecular mechanisms by which *atrogin-1* contributes to hypertrophy via transcription remain to be elucidated. The present results showed that many genes significantly altered in cell development and hypertrophic cardiomyopathy by overexpressed *atrogin-1*, Myh6, Serca2 (ATP2A2) and Prkcz were significantly up-regulated, whereas Itga3 (also known as CD49c) and Foxa2 were down-regulated.(Table 4). With respect to hypertrophic cardiomyopathy, Serca2 and Myh6 are major determinants of both cardiac relaxation and contraction. The Serca2 is of central importance for refilling of the sarcoplasmic reticulum (SR) Ca2^+^ store and cardiac contractility. Deletion of Serca2 function is associated with HF in animal models [39], whereas lentivirus vector-mediated Serca2 gene transfer ameliorates HF induced by myocardial infarction in rat [40]. In the heart, two isoforms of myosin heavy chain (MHC), Myh6 (also known as α-MHC) and Myh7 (also known as β-MHC), exist in the mammalian ventricular myocardium, and α-MHC is highly expressed in adult cardiomyocytes, whereas β-MHC is expressed in embryonic cardiomyocytes. Cardiac stress triggers adult hearts to undergo hypertrophy and a shift from Myh6 to fetal Myh7 expression. Furthermore, LPS is known to directly induce cardiomyocyte hypertrophy through activation of calcineurin signaling pathway [49], whereas overexpression of *atrogin-1* promotes calcineurin degradation and inhibits its activity, resulting in inhibition of cardiac hypertrophy [5]. Consistent with our previous data [5], [6], the present study showed that overexpression of *atrogin-1* inhibits LPS-induced cardiomyocyte hypertrophy, decreased the expression of hypertrophic marker ANF and up-regulated the level of Myh6 expression in cardiomyocytes. Thus, these results further confirmed that atrogin-1 exerts an important role in controlling cardiomyocyte hypertrophy.

Functional annotation of the *atrogin-1*-infected cardiomyocyte signature analysis led us to three main pathways: metabolic pathways, inflammation signaling pathways and hypertrophic cardiomyopathy. There is a substantial body of work linking metabolic and inflammatory pathways with cardiovascular diseases including hypertrophy and HF [60], [61]. Changes in mitochondrial gene expression were evident, ranging from the up-regulation of genes involved in energy metabolism, such as fatty acid biosynthetic process and ATP synthesis, and those important in maintaining energy metabolic pathways, such as Ptgis, Pycr1 and Lsyna1, SLC33A1 (Table 5). Ptgis mediates prostaglandin biosynthetic process and fatty acid biosynthetic process [34], [62]. Pycr1 encodes an enzyme involved in the cellular response to oxidative stress and amino acid biosynthetic process [35]. The Pycr1 protein is located in the mitochondria-the “power houses” of the cell that provide energy for cell's consumption [35]. Lsyna1 encodes a rate-limiting enzyme in the synthesis of all inositolcontaining compounds [63]. SLC33A1 functions as an acetyl-CoA transporter that is required for the formation of O-acetylated (Ac) gangliosides [64], [65]. These data indicate that *atrogin-1* plays an important role in regulating cardiomyocyte metabolic pathway.

It is well known that LPS directly induces inflammation in various cell types [1], [2]. We then determine if *atrogin-1* inhibits LPS-induced proinflammatory response in cardiomyocytes. *Atrogin-1* overexpression markedly inhibited the expression of proinflammatory-related genes including IL-1β, IL-6, Lbp, Ptgs2 and Serpinb2, Cxcl1, Cxcl2 and Ccl20 in cardiomyocytes, but increased anti-inflammatory gene IL-1r1 expression compared to Ad-GFP control under basal condition or in response to LPS stimulation (Figure 5A, Table 5), whereas these effects were reversed by knockdown of *atrogin-1* by siRNA (Figure 5B, Table 5). These results indicate that increased expression of *atrogin-1* inhibits cardiomyocyte inflammation.

The MAPK and NF-κB signaling pathways have been identified as the crucial regulators of cardiomyocyte apoptosis, cardiac hypertrophy and inflammation in response to various injury [66], but it is unknown if atrogin-1 affects activation of MAPK and NF-κB signal pathways in cardiomyocytes in response to LPS. Recently, a study demonstrated that depletion of *atrogin-1* inhibits cardiac hypertrophy in part through stabilization of IκB-α and inactivation of NF-κB [12]. To further investigate the mechanisms of *atrogin-1* in LPS-induced cardiac injury, we examined activation of ERK, JNK, p38 and p65/NF-κB signaling pathways. Under basal condition, overexpression of *atrogin-1* decreased the levels of ERK, JNK, p38 and p65/NF-κB phosphorylation compared with Ad-GFP control (Figure 6A), suggesting that *atrogin-1* may be involved in regulation of MAPK and NF-κB activation. After stimulation of LPS, the levels of ERK, JNK, p38 and p65/NF-κB phosphorylation were markedly increased, whereas these effects were markedly attenuated by atrogin-1 infection in cardiomyocytes (Figure 6A). In contrast, knockdown of endogenous *atrogin-1* in cardiomyocytes had opposite effects (Figure 6B). Thus, *atrogin-1* exerts its inhibitory effects on cardiomycoyte survival, hypertrophy and inflammation via inhibition of MAPKs and NF-κB pathways.

## Conclusion

The current study provides a comprehensive, global view of gene expression patterns induced by *atrogin-1* in the neonatal rat cardiomyocytes. Overexpression of *atrogin-1* results in marked alterations of gene expression profiles that are associated with cardiomyocyte survival, proliferation, inflammation, metabolism and hypertrophy, thereby leading to inhibition of cardiomycoyte survival, growth and hypertrophy under basal condition or in response to LPS stimualtion. The mechanisms underlying these effects were associated with inactivation of MAPK (ERK, JNK and p38) and NF-κB signaling pathways. These results may provide novel insight into how *atrogin-1* modulates the programming of cardiac muscle gene expression.

## Materials and Methods

All procedures were approved by and performed in accordance with the Animal Care and Use Committee of Capital Medical University (20110820).

### Neonatal rat cardiomyocytes isolation and culture

Primary cardiomyocytes were prepared by enzymatic disassociation of 1- to 2-day old Sprague-Dawley rats as described previously [5]. The isolated cardiomyocytes were resuspended in fresh DMEM/F12 containing 10% fetal bovine serum (FBS) and 1% penicillin-streptomycin and were plated into the Laminin (Sigma) pre-coated dishes (Corning) and incubated for 24 h at 37°C, then cultured with serum-free DMEM/F12 in later experiments.

### Adenovirus Infection

Twenty-four hours after plating, neonatal rat cardiomyocytes were infected with various adenovirus vectors as indicated and cultured with serum-free DMEM/F12 for 24 hours. Recombinant adenoviruses expressing GFP alone (Ad-GFP), *atrogin-1* (Ad-*atrogin-1*-GFP), siRNA-control or siRNA-*atrogin-1* were generated using the AdEasy system (MP Biomedicals Inc.) as described previously [18]. Fluorescent images were collected on a fluorescence microscope (Nikon TE 2000-U, Japan). The infection efficiency of adenoviruses was determined by counting the number of cells with green fluorescent protein (GFP).

### RNA isolation and GeneChip processing

Total RNA was isolated with TRIzol (Invitrogen) from neonatal rat cardiomyocytes in three independent experiments according to manufacturer's instructions. The quantity and purity of all RNA samples was determined using the Nanodrop ND-1000 spectrophotometer (Thermo Fisher, Waltham, MA, US), and RNA integrity was determined with the Bioanalyzer 2100 (Agilent technologies, Santa Clara, CA, US). Five micrograms of total RNA was amplified with the GeneChip One-Cycle cDNA synthesis Kit (Affymetrix) and GeneChip IVT labeling Kit (Affymetrix). Fifteen micrograms biotin-labeled complementary RNA (cRNA) was fractionated and hybridized to Affymetrix GeneChip Rat Genome 230 2.0 array according to the manufacturer's instructions. After16 h of hybridization, the Gene Chips were washed and stained on a Fluidics Station 450 (Affymetrix) and scanned in a confocal scanner (Affymetrix GeneChip Scanner 3000) according to the Affymetrix GeneChip Expression Analysis Manual as described previously [67]. On the GeneChip Rat Genome 230 2.0 array, 31,000 probe sets analyze the expression of 30,000 transcripts representing 28,000 known genes.

### Microarray data analysis

Analysis of microarray data was performed as described previously [67]. Briefly, raw intensities from the CEL files were analyzed using GeneSpring GX 11.0 (Agilent Technologies) to generate robust multi-array average (RMA) intensity in log_2_ scale for each probe set. Differentially expressed genes in Ad-*atrogin-1*-GFP group compared to Ad-GFP control group were detected with two statistical tests, a statistical technique used to compare means of two samples implemented in GeneSpring GX 11.0. For each transcript, fold change, statistical significance of differential expression and false discovery rate (FDR) were calculated. Fold change was calculated using the average signal from each experimental group. The recovered P-values of the comparisons were then corrected using a step-up false negative/positive rate value of 5%. The resulting list of significantly differentially expressed genes was filtered to include only genes that demonstrated 2-fold or greater up- or down-regulation. To search for enrichment of specific biological processes, the genes showing significantly differential expression between the two groups were classified into functional groups according to GO (Gene Ontology). As input, the differentially regulated probe sets from the comparison were used. As output, significant processes (GOTERM) with fold changes ≥2-fold or ≤−2-fold were generated. Furthermore, pathway analysis for the comparison was conducted through the use of KEGG Pathways database, which is a bioinformatics resource for linking genomes to life and the environment. Details can be found at http://www.genome.jp/kegg/. The microarray data discussed in this publication have been deposited in NCBI's Gene Expression Omnibus and are accessible through GEO Series accession number GSE31117 (http://www.ncbi.nlm.nih.gov/geo/query/acc.cgi?acc=GSE31117).

### Real-time quantitative PCR analysis

Microarray results for the expression profiling experiments were verified by real-time quantitative RT-PCR (qRT-PCR) using a Bio-Rad iQ5 Real-Time PCR detection system, employing β-actin as the endogenous control gene as described previously [67] . All reactions were conducted in triplicates and the data was analyzed using the delta delta Ct (**ΔΔ**Ct) method [68]. Oligonucleotide primers for qRT-PCR were designed using the Primer 5.0 software and the primers were designed to span large introns to eliminate possible contaminating genomic DNA. Rat gene-specific oligonucleotide primers used for qRT-PCR analyses are listed in Table 6.

**Table 6:** Primers used for Quantitative realtime RT-PCR Analyses.

Genes	Forward primer	Reverse primer
Axin2	5′-GCGTGAGATCCACAGAAACG-3′	5′-TCGCTGGATAACTCGCTGTC-3′
Calr	5′-CCGATGCGAATATCTATGCC-3′	5′-TCATTGCCAAACTCCTCTGC-3′
Dkk2	5′-CATGAACCAAGGACTGGCTT-3′	5′-CAGGCTGAAGATCCTTGGTG-3′
Il1r1	5′-TTGTCTCATTGTGCCTCTGC-3′	5′-TAAGAGGACAGCTGCGAATG-3′
CyclinD2	5′-TCAAGTGCGTGCAGAAGGAC-3′	5′- TGGCCAGAGGAAAGACCTCT -3′
MMP3	5′-TCCTTCGATGCAGTCAGCAC-3′	5′-TGTTGGATGGAAGAGACGGC-3′
Cadm1	5′-CACAGGTGATGGGCAGAATC-3′	5′-TGCCTGTTGGGGTTCAGTAG-3′
Cxxl1	5′-CCACACTCAAGAATGGTCGC-3′	5′-GTTCACCAGACAGACGCCAT-3′
Cxcl2	5′-TTTGTCTCAACCCTGAAGCC-3′	5′-TGAGGTACAGGAGCCCATGT-3′
Cxcl6	5′-TCCTGCTCGTCATTCACCCT-3′	5′-CAAACACAGCGTAGCTCCGT-3′
Il-6	5′-TCTGCTCTGGTCTTCTGGAG-3′	5′-TTGCTCTGAATGACTCTGGC-3′
Il-1β	5′-CTCTGTGACTCGTGGGATGATG-3′	5′-CCACTTGTTGGCTTATGTTCTGTC-3′
Ptgs2	5′-GATCACATTTGATTGACAGC-3′	5′-TCCTTATTTCCTTTCACACC-3′
Serpinb2	5′-TCCTTGGTGCTCAGGCTAAC-3′	5′-CAGCCATGGAAGTTCTCTGG-3′
Ccl20	5′-ACCTCCTCAGCCTAAGAACCAAGA-3′	5′-TGTGCAGTGATGTGCAGGTGAAAC-3′
Myh7	5′-CGAGGCAAGCTCACGTATAC-3′	5′-CTTGGCTTCTGTTTCCTCCT-3′
ANF	5′-CTTCTCCATCACCAAGGGCTT-3′	5′-GGATTTGCTCCAATATGGCCT-3′
serca2	5′-CAGTTCATCCGCTACCTCATCTCC-3′	5′-CGCAGTGGCAGGCAGACC-3′
GADPH	5′-CCCCCAATGTATCCGTTGTG	TAGCCCAGGATGCCCTTTAGT
β-actin	5′-GGAGATTACTGCCCTGGCTCCTA-3′	5′-GACTCATCGTACTCCTGCTTGCTG-3′

### Western blot analysis

Cardiomyocytes were lysed in lysis buffer (50 mM Tris-HCl, pH 7.5, 1 mM DTT, 150 mM NaCl, 0.5% NP-40, 1 mM EDTA, 1 mM PMSF, 1 mM Na3VO4, 1 mM NaF, plus protease inhibitor cocktail). Forty micrograms of total proteins was separated by SDS-PAGE, transferred to PVDF membranes (Millipore), and analyzed by western blot with anti-atrogin-1 antibody (H300, Santa Cruz) as described previously [5], using β-actin (I-19, Santa Cruz) as the internal control. Protein levels were quantified by using Gel-pro 4.5 Analyzer (Media Cybernetics).

### TUNEL assays

Primary cardiomyocytes were infected with Ad-*atrogin-1* or Ad-GFP control (MOI = 10) for 24 hours and then treated with 10 ng/ml LPS (Sigma) for 24 hours. TUNEL assays were performed using the In Situ Cell Death Detection Kit, TMR red (Roche) according to manufacturer's instructions. Apoptosis was evaluated by Laser scanning confocal fluorescence microscope (Leica TCS SP2, Germany). Quantitation of cardiomyocyte apoptosis was performed based on the percentage of TMR red labeled nuclei with image-pro plus 6.0 software.

### Measurement of cardiomyocyte hypertrophy

Primary cardiomyocytes were infected with Ad-*atrogin-1* or Ad-GFP control (MOI = 10) for 24 hours and then treated with PBS or lipopolysaccharides (LPS, 1 µg/ml) for 8 hours, then cells were fixed and stained with anti-α-actinin antibody followed by Alexa Fluor 568-conjugated goat anti-mouse IgG (red), and nuclei were stained with DAPI (blue). A representative field is shown for each condition. A minimum of 100 randomly chosen cells measured in each group. Quantitation of cell surface area was performed as described previously [5].

### Statistical analysis

Data for qRT-PCR analyses were presented as means ± SEM. Differences between groups were evaluated for statistical significance using a two-tailed Student's *t* test with GraphPad Prism 5.0 or Stata/SE 10.0 softwares. *P* values less than 0.05 were regarded as significant.
