# Correction: Transcriptional Effects of E3 Ligase *Atrogin-1*/*MAFbx* on Apoptosis, Hypertrophy and Inflammation in Neonatal Rat Cardiomyocytes

**DOI:** 10.1371/annotation/97e1be8b-a068-4e4b-9e8d-14315bb34e7e

**Published:** 2013-07-03

**Authors:** Yong Zeng, Junjie Li, Hong-Xia Wang, Shu-Bin Guo, Hui Yang, Xiang-Jun Zeng, Quan Fang, Chao-Shu Tang, Jie Du, Hui-Hua Li

The author list for this article should be updated to include Junjie Li as co-first author for the work. The revised author list should thus read as follows:

Yong Zeng*, Junjie Li*, Hong-Xia Wang, Shu-Bin Guo, Hui Yang, Xiang-Jun Zeng, Quan Fang, Chao-Shu Tang, Jie Du, Hui-Hua Li

*These authors contributed equally

Junjie Li is affiliated at the Department of Pathology, Institute of Basic Medical Sciences, Chinese Academy of Medical Sciences and Peking Union Medical College, Tsinghua University, Beijing 100005, China.

Junjie Li declares no competing interests in relation to this work and contributed to the following aspects of the study: Performed experiments, analyzed the data, wrote the paper, contributed analysis and tools.

The correct citation is: Zeng Y, Li J, Wang H-X, Guo S-B, Yang H, Zeng X-J, et al. (2013) Transcriptional Effects of E3 Ligase Atrogin-1/MAFbx on Apoptosis, Hypertrophy and Inflammation in Neonatal Rat Cardiomyocytes. PLoS ONE 8(1): e53831. doi:10.1371/journal.pone.0053831. The correct abbreviation in Contributions is: JL. 

