# Correction: Transcriptional Effects of E3 Ligase *Atrogin-1/MAFbx* on Apoptosis, Hypertrophy and Inflammation in Neonatal Rat Cardiomyocytes

**DOI:** 10.1371/journal.pone.0267947

**Published:** 2022-04-27

**Authors:** Yong Zeng, Hong-Xia Wang, Shu-Bin Guo, Hui Yang, Xiang-Jun Zeng, Quan Fang, Chao-Shu Tang, Jie Du, Hui-Hua Li

There is an error in the Ad-GFP panel in Fig 1A in this article [1]. Specifically, the panel for Ad-GFP is incorrect and shows an image that overlaps with the panel for Ad-*atrogin-1* in Fig 1A.

**Fig 1:**
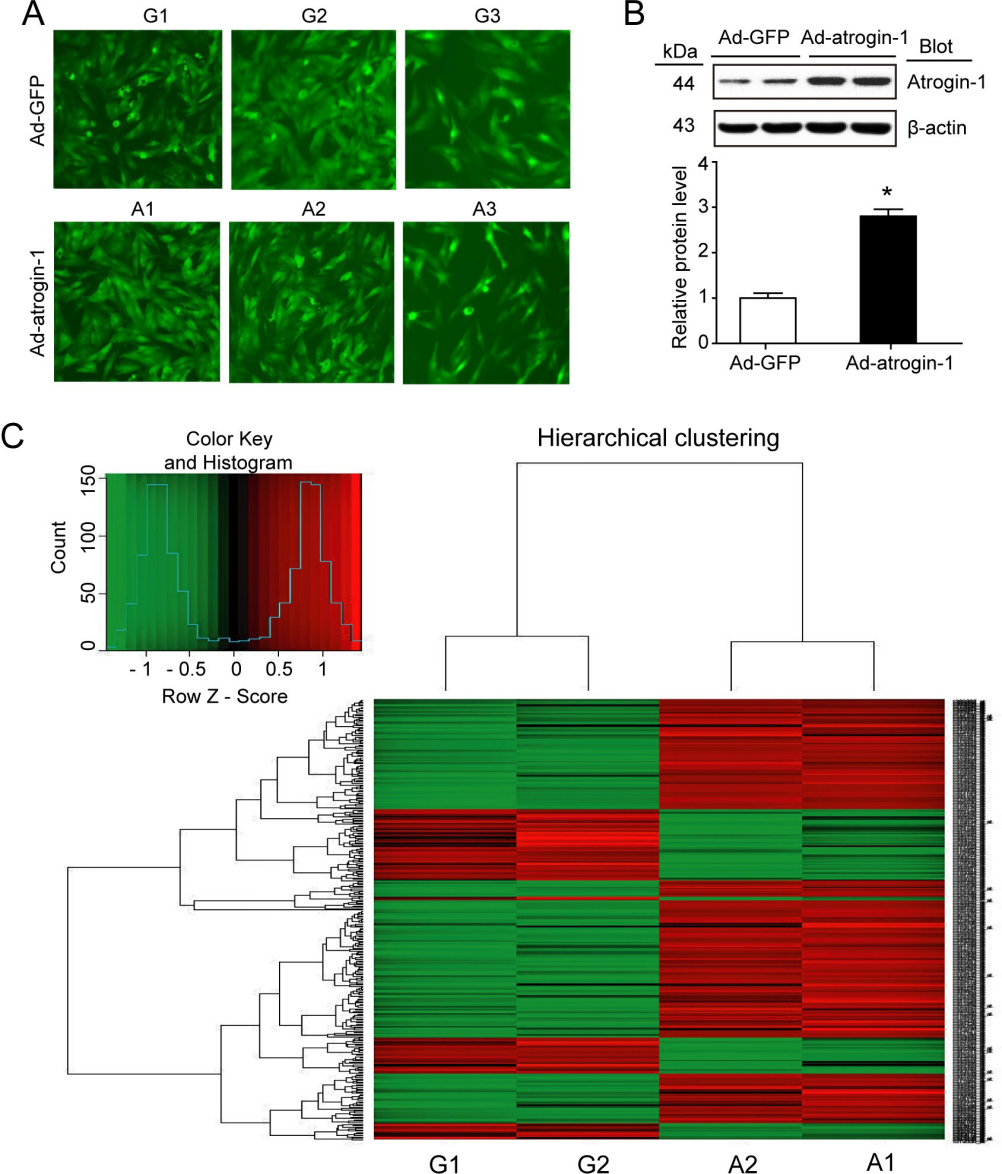
Infection of adenovirus *atrogin-1* and microarray analysis. **A**. Neonatal rat cardiomyocytes were infected with adenovirus green fluorescent protein (GFP) control (Ad-GFP; G1, G2 and G3) or *atrogin-1* (Ad-*atrogin*-*1*-GFP; A1, A2 and A3). The degree of infection for each group was visualized for GFP 24 hours later using fluorescence microscopy (Magnification, x400). **B**. Cardiomyocytes (n = 3 per group) were collected. A half of samples was lysed with RIPA buffer, and equal amount of proteins were determined by Western blot analysis with anti-*atrogin-1*, using β-actin as the internal control (top). Quantitative analysis of the relative protein bands was shown (bottom, n = 3). Data represent the mean±SEM. *P < 0.05 vs. Ad-GFP control. **C**. Since the infection efficiency and protein levels of *atrogin-1* were similar among three samples. Equal amount of total RNA (5 μg) isolated from another half of samples (G1, G2, and A1, A2) using TRIzol reagent was used for GeneChip assay. Hierarchical clustering depicting expression profiles of differentially expressed genes in Ad-*atrogin-1* (A1 and A2) and Ad-control (G1 and G2) groups. Data from individual sample are shown. A subset of genes displays significant expression changes at ≥2-fold or ≤2-fold. Gene expression levels are shown as color variations (red: up-regulated expression; green: down-regulated expression).

Here the authors provide a revised version of Fig 1 in which the Ad-GFP panel has been replaced and two additional replicate images captured during the original experiments for both Ad-GFP (G2 and G3) and Ad-*atrogin-1* (A2 and A3) have been included. Underlying data supporting the revised Fig 1A are in S1 File.

The revised Fig 1 also includes an additional chart for the expression level of *atrogin-1* by western blot in Fig 1B. Underlying quantitative data tables and additional replicate western blots from the original experiments underlying the chart in the revised Fig 1B are in S2 and S3 File, respectively.

The authors provide the following additional methodological information for the experiments reported in Fig 1: Cardiomyocytes were infected with Ad-GFP (n = 3 per group; G1, G2, and G3) or Ad-*atrogin-1* (n = 3 per group; A1, A2, and A3) for 24 hours, and then the degree of infection was visualized using fluorescence. Half of the cells in each group were lysed with RIPA buffer, and total RNA was isolated from the other half using TRIzol reagent for GeneChip assay (G1, G2 and A1, A2). An equal amount of cell extracts were analyzed using western blots with anti-*atrogin-1* or β-actin antibodies. The membranes were cut into smaller pieces for incubation of the antibodies after protein transfer from the gel. *Atrogin-1* and the β-actin controls in Fig 1B and S3 File were run on separate gels. Individual *atrogin-1* protein levels were quantified by densitometry and normalized to the corresponding β-actin level, and the value for Ad-*atrogin-1* protein level was expressed relative to the Ad-GFP control after normalization.

The authors apologize for the errors in the published article and confirm that the corrections to Fig 1 do not affect the validity of the data or the overall conclusions.

Underlying data supporting other results in the published article are available from the corresponding author.

## Supporting information

S1 File Six original fluorescent images. (TIF)

S2 File Data for Fig 1B bottom chart. (XLSX)

S3 File Updated Fig 1B original blot images. (TIF)
