# Supplementary figures and images for: Correction: Transcriptional Effects of E3 Ligase Atrogin-1/MAFbx on Apoptosis, Hypertrophy and Inflammation in Neonatal Rat Cardiomyocytes
Source: PLoS One. 2022 Apr 27;17(4):e0267947. doi: 10.1371/journal.pone.0267947 (PMC9045633; doi:10.1371/journal.pone.0267947)

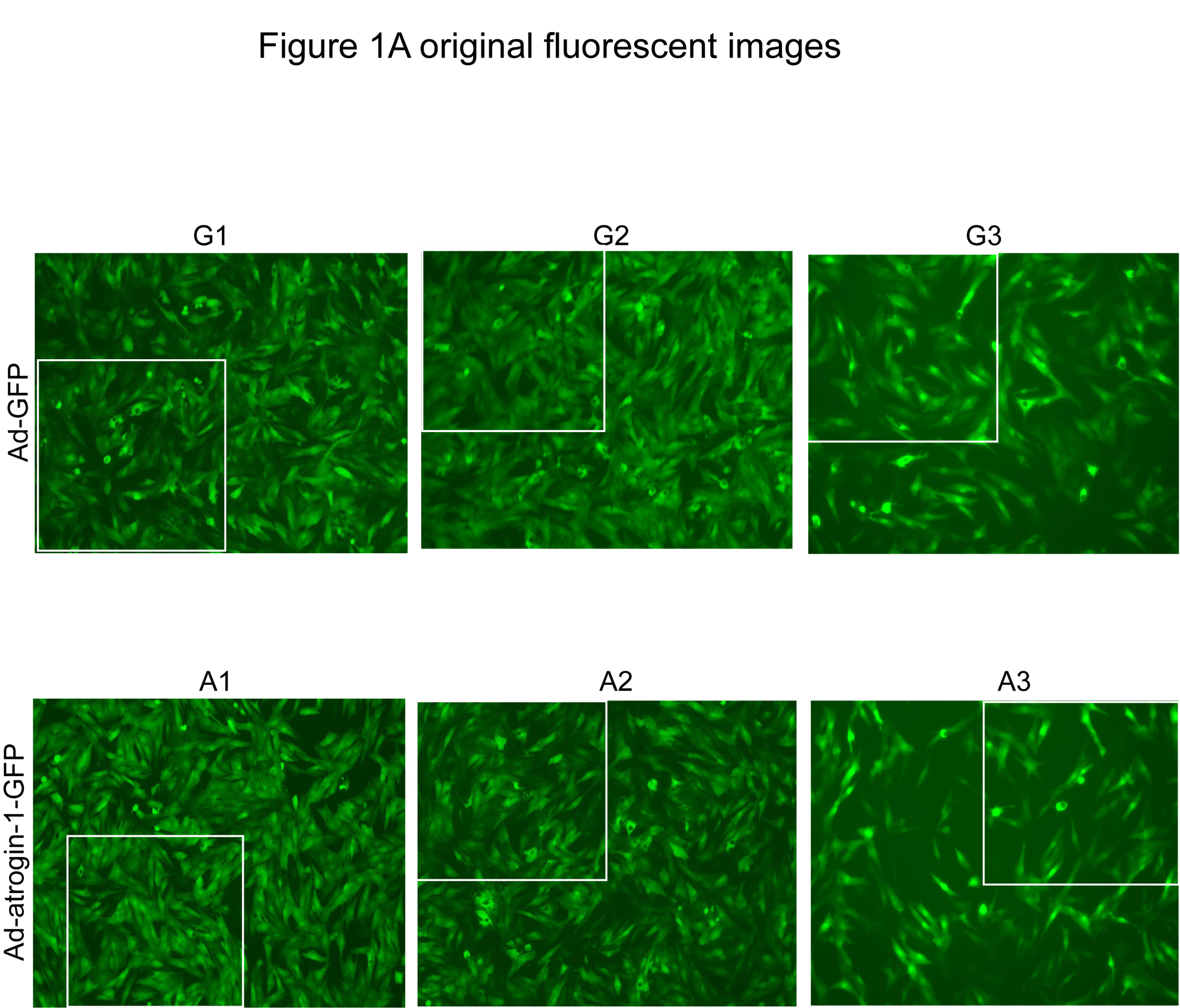

Supplement: S1 File — (TIF) [file pone.0267947.s001.tif]

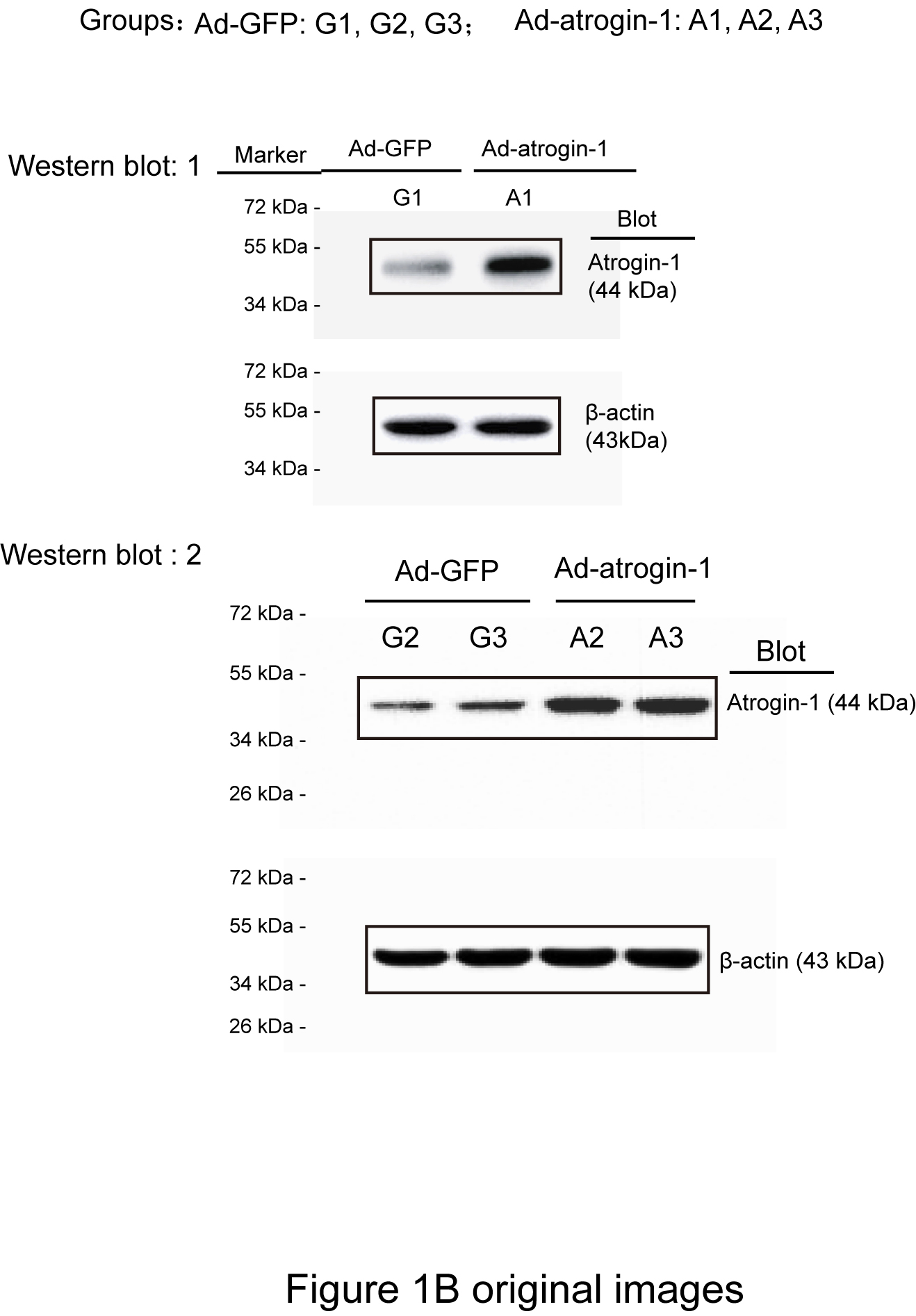

Supplement: S3 File — (TIF) [file pone.0267947.s003.tif]
